# The Emerging Role of IL-9 in the Anticancer Effects of Anti-PD-1 Therapy

**DOI:** 10.3390/biom13040670

**Published:** 2023-04-12

**Authors:** Daria Vinokurova, Lionel Apetoh

**Affiliations:** 1 UMR 1231, Lipides Nutrition Cancer, INSERM, 21000 Dijon, France; 2 UFR des Sciences de Santé, Université de Bourgogne, 21000 Dijon, France; 3 Brown Center for Immunotherapy, Indiana University Melvin and Bren Simon Comprehensive Cancer Center, Indiana University School of Medicine, Indianapolis, IN 46202, USA

**Keywords:** interleukin 9, anti-PD-1, T_H_9 cells, cancer immunotherapy

## Abstract

PD-1 blockade rescues failing anticancer immune responses, resulting in durable remissions in some cancer patients. Cytokines such as IFNγ and IL-2 contribute to the anti-tumor effect of PD-1 blockade. IL-9 was identified over the last decade as a cytokine demonstrating a potent ability to harness the anticancer functions of innate and adaptive immune cells in mice. Recent translational investigations suggest that the anticancer activity of IL-9 also extends to some human cancers. Increased T cell-derived IL-9 was proposed to predict the response to anti-PD-1 therapy. Preclinical investigations accordingly revealed that IL-9 could synergize with anti-PD-1 therapy in eliciting anticancer responses. Here, we review the findings suggesting an important contribution of IL-9 in the efficacy of anti-PD-1 therapy and discuss their clinical relevance. We will also discuss the role of host factors like the microbiota and TGFβ in the tumor microenvironment (TME) in the regulation of IL-9 secretion and anti-PD-1 treatment efficacy.

## 1. Introduction: The Success of PD-1 Blockade Relies on Effector Cytokines

Tumor-reactive T cells can become dysfunctional due to chronic exposure to tumor-associated antigens and immune-suppressive stimuli in the tumor microenvironment (TME) [1]. Rescuing T cell responses in tumors using immune checkpoint blockade has allowed durable remissions even in patients with metastatic cancers. One survival analysis revealed long-lasting benefits of PD-1 blockade: estimated 5-year survival rates after nivolumab treatment were markedly higher compared with conventional cancer therapies [2]. However, our understanding of how PD-1 blockade reinvigorates anti-tumor immune responses is still elusive. As a substantial fraction of patients fail to develop a long-term response to PD-1 blockade, deeper insights into the effects of PD-1 blockade on immune responses in tumors are crucial.

CD8 T cells recognize tumor antigens, and cytotoxic responses mediated by CD8 T cells are considered a major mechanism of the anti-cancer activity of PD-1 blockade. Rescuing tumor-reactive CD8 T cells does not, however, encompass all the immune events contributing to the clinical efficacy of anti-PD-1 therapy. Cytokines play a crucial role in anti-tumor immunity by orchestrating the activation, differentiation, and expansion of various immune cells. One of the cytokines contributing to the anti-tumor response induced by PD-1 blockade is IFNγ. This cytokine induces cell cycle arrest and apoptosis of tumor cells and amplifies anti-tumor immune responses through the activation of different effector cell types such as dendritic cells (DCs), macrophages, and T cells [3]. In cancer patients, an increase in IFNγ expression and IFNγ-related transcriptomic profile predicts the response to anti-PD-1 therapy [4,5]. PD-1 blockade can stimulate the production of IFNγ in the TME, and not only by CD8 T cells [6]. IFNγ promotes the anti-cancer efficacy of PD-1 blockade in preclinical models including pancreatic cancer, which is generally resistant to anti-PD-1 therapy [7]. Besides IFNγ, other cytokines contribute to the anti-PD-1-driven anti-tumor response. PD-1/PD-L1 blockade (alone and in combination therapies) restored IL-2 production by tumor-infiltrating T cells which was associated with tumor inhibition [8,9]. An agonist of the IL-2 pathway in combination with nivolumab is being currently assessed in clinical trials for multiple human cancers as it demonstrated promising results in a phase I trial regarding clinical response and activation of tumor-infiltrating CD8 T cells [10].

## 2. Human IL-9^+^ Cells in Cancer and Associations with the Response to Anti-PD-1

IL-2 regulates CD4 T cell differentiation via the IL-2/STAT5 axis. IL-2/STAT5 signaling promotes the differentiation of IL-9-producing CD4 T cells—T_H_9 cells. This subset of CD4 T cells was identified in 2008 [11,12]. The ability of T_H_9 cells to produce IL-9 is greatly attenuated in the absence of IL-2 or STAT5 [13,14]. STAT5 binds to the *Il9* promoter, which is necessary for the demethylation of the *Il9* locus and its accessibility to other transcription factors [15]. IL-9 is a pleiotropic cytokine playing a range of functions in the immune system. During the last decade, multiple studies have revealed the contribution of IL-9 to tumor immunity. IL-9 has opposing effects on IL-9R-positive tumor cells: from cytostatic and cytotoxic effects [16] to promoting tumor cell growth and migration [17,18]. IL-9 can also potently promote an anti-tumor immune response in different murine models [19]. IL-9 can directly or through DCs promote CD8 T cell responses [20,21]. IL-9 can also trigger innate responses in tumors by activating mast cells [22,23]. In addition to T_H_9 cells, other IL-9-producing cell types include CD8 T cells (Tc9), regulatory T cells, Vδ2 T cells, innate lymphoid cells type 2 (ILC2s), mast cells, and natural killer (NK) cells [24]. In human cancers, IL-9-producing cells have been detected in a growing list of malignancies including breast [21], lung [25], and colon cancer [26] (Table 1).

IL-9 has recently been linked to response to anti-PD-1 treatment. The first study which reported the correlation between IL-9 and the outcome of anti-PD-1 therapy was designed by Nonomura et al. in 2016 [34] (Table 2). The authors examined different T-cell subsets and cytokines in a cohort of 46 melanoma patients before and after anti-PD-1 treatment (nivolumab). The treatment increased the frequency of one CD4 T-cell subset in peripheral blood—T_H_9 cells. Furthermore, the increased frequency of circulating T_H_9 cells was significantly associated with the response to the treatment. The frequencies of other CD4 T-cell subsets, CD8 T cells, or IFNγ levels were not associated with the response to PD-1 blockade.

A study in muscle-invasive bladder cancer provided further evidence of the predictive value of IL-9-producing cells in response to anti-PD-1 treatment [30]. This multicenter study obtained data from 259 patients from two independent clinic centers. It showed that CD8 T cells responded to nivolumab treatment by expansion and production of effector molecules only in patients with high IL-9^+^ cell infiltration. Accordingly, nivolumab treatment suppressed growth and induced apoptosis of tumor cells only in the high IL-9 group. Without immunotherapy, infiltration with IL-9^+^ cells was, however, associated with decreased overall and disease-free survival and progressive dysfunction of CD8 T cells. The discrepancy could be due to the association of IL-9^+^ cell infiltration with high PD-1 expression of CD8^+^ T cells. Together, these data suggest IL-9^+^ cell infiltration may be a predictive marker of response to anti-PD-1 treatment. In addition, infiltration with IL-9^+^ cells might reflect the presence of an anti-tumor immune response actively suppressed through the PD-1/PD-L1 pathway.

Findings of a recent study in gastric cancer patients show that IL-9 could help restore CD8 T cell function upon PD-1 blockade [32]. The authors demonstrated that recombinant IL-9 (rIL-9) enhanced the anti-cancer activity of anti-PD-1 treatment (pembrolizumab) ex vivo by inducing a cytotoxic response in CD8 TILs. The anti-tumor effect of rIL-9 was abrogated in the absence of CD8 T cells. Analysis of tumor samples from 453 patients showed that most CD8 TILs were IL-9R^+^ and IL-9 expression in TME was correlated with elevated CD8 T-cell infiltration and function, as illustrated by elevated secretion of effector molecules (IFNγ, perforin, granzyme) and reduced expression of inhibitory receptors. These results support previous findings indicating that IL-9 enhances CD8 T cell responses in tumors and suggest that IL-9 can synergize with anti-PD-1 therapy in reinvigorating CD8 T cell responses.

## 3. T_H_9 Cells in the Immune Response Elicited by PD-1 Blockade

The studies discussed above documented that IL-9-producing cells were associated with the response to anti-PD-1 treatment in cancer patients and suggested that IL-9 could be involved in the anti-tumor immune response elicited by this immunotherapy. A recent paper by Feng et al. provided additional evidence that IL-9 enhanced the response to anti-PD-1 treatment [36]. The authors found that treatment with rIL-9 promoted the response to anti-PD-1 treatment in a mouse model of IL-9 receptor-positive lung cancer, CMT167. Several mechanisms of the anti-tumor function of IL-9 could contribute to the observed synergy with anti-PD-1 treatment [19]. Feng et al. found that although the CMT167 tumor cells were IL-9R positive, IL-9 did not affect the viability of these cells. However, IL-9R knockdown in CMT167 cells abrogated the IL-9-driven tumor growth inhibition and enhanced T-cell infiltration. This indicates that in this model, IL-9 induced an anti-tumor immune response through action on tumor cells, rather than through immunomodulation. The study revealed that IL-9 stimulation increased MHC I expression in tumor cells in an ERK-dependent manner, which promoted tumor infiltration with T cells. Additionally, IL-9 increased PD-L1 expression in tumor cells and PD-1 expression in T cells which explained the synergistic effects observed upon PD-1 blockade. This study therefore demonstrated that IL-9 augmented the response to anti-PD-1 treatment. Unlike in other mouse tumor models such as melanoma, the underlying mechanisms here included regulation of MHC I and PD-L1 expression in tumor cells and PD-1 expression on TILs.

The potential role of T_H_9 cells in anti-PD-1-induced anti-tumor immune response may extend to other cytokines beyond IL-9. T_H_9 cells also secrete IL-21—a pleiotropic cytokine that among other activities enhances the cytotoxicity of CD8 T cells and NK cells [37]. IL-21 released by IL-1β-stimulated T_H_9 cells induced IFNγ secretion from CD8 and NK cells in tumor-bearing mice, resulting in tumor growth inhibition [38]. Human T_H_9 cells also promoted the cytotoxic function of CD8 T cells against autologous tumor cells in an IL-9- and IL-21-dependent manner [21]. IL-21 has recently emerged as a cytokine promoting the antitumor effects of immune checkpoint blockade in preclinical models [39]. IL-21 tumor cell-targeting fusion protein was shown to induce the expansion of functional tumor-reactive CD8 T cells (PD-1^int^Tim-3^–^CD8^+^) and promote the formation of memory T cells [40]. The IL-21 fusion protein inhibited tumor growth and demonstrated significant synergy with PD-1 blockade in advanced tumors. In another study, CD4 T cell-derived IL-21 induced the expansion of strongly cytolytic CX3CR1^+^ CD8 TILs, which promoted tumor control [41]. In the absence of CD4 T-cell help, anti-PD-L1 failed to promote the expansion of these cytolytic CD8 T cells. IL-21 also enhanced the anti-tumor effect of combined PD-1/Tim-3 blockade in MHC I-deficient tumors by reinvigorating tumor-infiltrating NK cells [42]. Altogether, IL-21 can potently promote anti-tumor immune responses elicited by PD-1 blockade via several mechanisms. Although this remains to be experimentally tested, T_H_9-derived IL-21 may therefore contribute to the effects of T_H_9 cells upon anti-PD-1 treatment.

## 4. The Role of the PD-1 Signaling in the Regulation of IL-9 Expression

**Figure 1:**
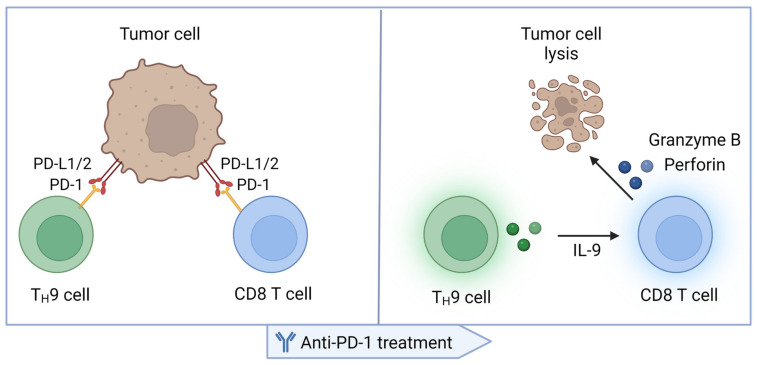
The proposed role of IL-9 in the response to anti-PD-1 therapy. IL-9 production in TILs is regulated by PD-1 signaling [26,34]. Anti-PD-1 antibody stimulates IL-9 production in TILs which enhances the cytotoxic properties of tumor-reactive CD8 T cells. This model is supported by observations in cancer patients: CD8 TILs of patients with high IL-9^+^ cell infiltration respond to nivolumab treatment by expanding and producing effector molecules [30].

The increase in circulating T_H_9 cells observed upon nivolumab treatment in melanoma patients suggested that PD-1 blockade could unleash T_H_9 cell responses in cancer [34]. The blockade of PD-L1/PD-1 signaling in isolated peripheral blood mononuclear cells (PBMCs) in vitro indeed promoted T_H_9 cell differentiation upon treatment by increasing the fraction of IL-9^+^CD4^+^ T cells more than two times. The findings of another study supported the idea that IL-9^+^ cells in tumors can be regulated by PD-1 signaling [26]. When analyzing tumor samples from 20 patients with colorectal cancer, the authors found that IL-9^+^ TILs expressed higher levels of PD-1 than other populations of TILs. Engagement of PD-1 with PD-L1 inhibited IL-9 production in isolated TILs. These observations in cancer patients are consistent with results obtained in preclinical models where anti-PD-1 treatment of mice with hepatocellular carcinoma increased IL-9 concentration in peripheral blood [43]. Stimulation by another ligand of PD-1—PD-L2—was also reported to regulate T_H_9 differentiation and IL-9 production [44]. These data indicate that PD-1 signaling affects IL-9 release. Together with the known role of IL-9 in CD8 T cells in cancer, these studies suggest that IL-9 responses rescued by anti-PD-1 treatment contribute to the effect of anti-PD-1 therapy by promoting anti-tumor CD8 responses (Figure 1).

In addition to T cells, the PD-1 pathway can regulate IL-9 expression in other IL-9-producing cells such as ILC2s [45]. ILCs are tissue-resident innate immune cells that are characterized by similar morphology to lymphocytes but lack antigen receptors. They orchestrate immune responses by producing cytokines. ILC2s are induced in tissues by alarmins that include thymic stromal lymphopoietin (TSLP), IL-33, and IL-25 and produce the T_H_2- and T_H_9-related cytokines IL-4, IL-5, IL-13, and IL-9 [46,47,48]. Activation of pulmonary ILC2s in vivo with IL-33 induces high PD-1 expression in these cells [45]. PD-1 deficiency increased IL-9 expression in ILC2s more than twice and more moderately increased IL-13 and IL-5 expression. PD-1 signaling may thus be a mechanism regulating IL-9 production in different cell types. PD-1-driven regulation of IL-9 production by ILC2s may be relevant in cancer as PD-1^+^ ILC2s were detected in human cancer, and PD-1 blockade in combination with IL-33 enhanced the anti-tumor function of ILC2s [49].

PD-1 signaling changes the metabolic program of activated T cells by inhibiting glycolysis [50]. T_H_9 cell development relies on glycolysis, as underscored by investigations demonstrating that blocking glycolysis inhibited the differentiation of T_H_9 cells [51]. Mechanistically, transcription factor HIF1α involved in glycolysis in activated T cells was found to bind and transactivate the *Il9* promoter [52]. PD-1 signaling can therefore compromise IL-9 production in T_H_9 cells by inhibiting glycolysis. Interestingly, T_H_9 cells are more dependent on glycolysis compared to other CD4 T cell subsets, as indicated by higher levels of lactate produced by T_H_9 cells, and therefore can be more sensitive to PD-1-driven metabolic changes [51]. Like in T cells, PD-1 deficiency in ILC2s increased IL-9 production, which was associated with a metabolism shift toward glycolysis [45]. These metabolic changes were associated with the increased expression of the transcription factors STAT5, STAT6, and GATA3, which are all known to contribute to IL-9 production in T cells.

## 5. Regulation of IL-9 in the Tumor Microenvironment

**Figure 2:**
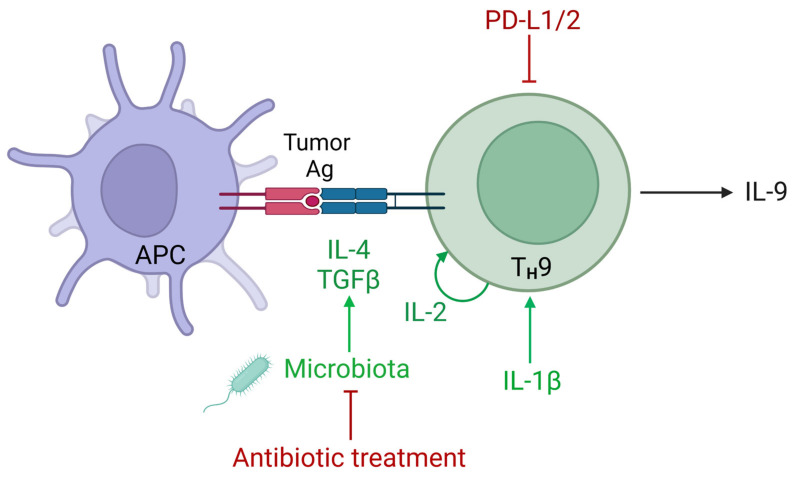
Regulation of IL-9 production in cancer. Key cytokines inducing IL-9 production by T cells—IL-4 and TGFβ—can be induced by the microbiota and their production can be compromised by antibiotic-driven dysbiosis [56]. IL-2 is another crucial cytokine for IL-9 production by T cells, it can act in an autocrine or paracrine manner [13,14]. IL-1β can promote IL-9 expression by T_H_9 cells in cancer [38] and induce T_H_9 cell differentiation in combination with IL-4 [57]. While TGFβ in combination with IL-4 induces IL-9 production by T cells, continuous TGFβ stimulation in cancer can restrict IL-9 production by CD4 T cells [53]. Continuous exposure to TGFβ can inhibit BFAR expression required for TGFβ signaling, therefore creating a negative feedback loop. Finally, PD-1 signaling restricts IL-9 production by T cells in cancer.

Different tumor cell-dependent and TME-dependent factors can contribute to IL-9 production and accordingly affect the response to anti-PD-1 treatment (Figure 2). TGFβ and IL-4 represent two cytokines inducing high IL-9 expression in CD4 T cells [11,12]. However, continuous exposure to high levels of TGFβ can impair IL-9 production in tumors [53]. The authors found that continuous exposure of CD4 T cells to TGFβ inhibits expression of bifunctional apoptosis regulator (BFAR), which is required for activation of TGFβ signaling, thereby forming a negative feedback loop restricting sustained TGFβ signaling. These cells had consequently impaired Smad2/3 phosphorylation and impaired IL-9 production under T_H_9 differentiation conditions. TGFβ can interfere with the response to PD-1/PD-L1 blockade [54,55]. Inhibition of IL-9 production by CD4 T cells could be one of the mechanisms of how TGFβ attenuates the responsiveness to PD-1 blockade. Pei et al. showed that blocking TGFβ in TME increased the response to PD-1 blockade, and it was associated with markedly increased concentrations of IL-9 in serum and infiltration of T_H_9 cells in tumors. This was not observed for other CD4 T cell subsets. Blocking IL-9 abrogated the beneficial effect of TGFβ neutralization on the response to anti-PD-1 treatment. This provided evidence that inhibition of IL-9 production could partially mediate the detrimental effect of TGFβ on the response to anti-PD-1.

T_H_9 cells can acquire a more inflammatory phenotype and stronger anti-tumor function in the absence of TGFβ [57]. Vegran et al. previously showed that the addition of IL-1β to the classical T_H_9 cell differentiation conditions—IL-4 and TGFβ—promoted the expression of IL-9 and IL-21 and enhanced the anti-tumor properties of T_H_9 cells [38]. Further work by Xue et al. revealed that while the addition of IL-1β to T_H_9 cells drove the upregulation of some genes, these cells largely resembled classical T_H_9 cells [57]. These results were in line with the findings of Vegran et al. who initially demonstrated that IL-1β enhanced but did not skew T_H_9 cell differentiation [38]. Omitting TGFβ to leave only IL-4 and IL-1β resulted in a generation of cells which still produced high levels of IL-9. Their transcriptional profile was, however, unique and differed from other CD4 T-cell subsets. T_H_9 cells generated in the absence of TGFβ markedly upregulated several pathways including the production of cytokines and cytolytic effector molecules such as granzymes. The expression of inhibitory receptors such as CTLA-4, PD-1, and LAG3 was conversely down-regulated compared with classical T_H_9 cells. These findings mirror previous results obtained upon differentiating naïve CD4 T cells into T_H_17 cells. While conventional T_H_17 cells are induced in presence of TGFβ and IL-6, subsequent studies found that a combination of IL-6 with IL-1β and IL-23 induced T_H_17 cell differentiation in the absence of TGFβ [58]. The latter T_H_17 cells acquired a more inflammatory phenotype as they did not produce IL-10 and gained the expression of IFNγ after adoptive transfer [58]. Overall, the cytokine environment in tumors can regulate the inflammatory potential of CD4 T cells such as T_H_9 and T_H_17 cells. The TGFβ-driven regulation of T_H_9 cell functions could attenuate the response to anti-PD-1. Because metastatic melanoma patients who responded to nivolumab treatment with an increase in T_H_9 cells in peripheral blood had higher levels of serum TGFβ [34], this regulation should be explored in different cancers.

The gut microbiota represents another factor that may shape responses to PD-1 blockade by regulating IL-9 responses [59]. A growing number of preclinical and clinical studies have established the microbiota as a critical factor regulating the efficacy of anti-PD-1 therapy in various cancers [60,61]. Three lines of evidence provided solid proof of this concept. The microbiota composition correlates with treatment outcomes, the administration of antibiotics attenuates the response to PD-1 blockade, and fecal microbiota transplantation shapes the response to treatment. The microbiota affects the response to anti-PD-1 treatment by controlling the recruitment and activation of various effector immune cells in tumors. Almeida et al. revealed that the microbiota drove the induction of T_H_9 cell responses in cancer [56]. The authors found that the colon of germ-free mice harbored markedly reduced levels of IL-9-producing T cells—both T_H_9 and Tc9 cells—compared with conventional mice. Similarly, dysbiosis induced by long-term antibiotic treatment resulted in a two-fold drop in IL-9-producing T cells in the colon. The observed decrease in IL-9-producing T cells was associated with reduced concentrations of IL-4 and TGFβ. This suggested that the microbiota supported the induction of IL-9^+^ T cells by inducing the release of these key T_H_9 cell-inducing cytokines. Next, the authors evaluated the impact of microbiota-driven T_H_9 responses in melanoma. Antibiotic treatment promoted the growth of melanoma lung foci and it was associated with decreased IL-9^+^ T-cell infiltration. Microbiota recolonization through fecal transplantation restored tumor control in antibiotic-treated mice to the level of control mice. Strikingly, injection of rIL-9 also largely restored tumor control compromised by the antibiotic treatment. It confirmed that in this melanoma model, tumor growth accelerated by disbalance in the microbiota was largely caused by IL-9 deficiency in the TME. The study of Almeida et al., therefore, revealed that the microbiota could play a critical role in the induction of T_H_9 cell responses. This affected melanoma growth. Because the microbiota controls the response to PD-1 blockade in various human cancers, these results further suggest that IL-9-producing T cells may mediate the effects of the microbiota in response to anti-PD-1 treatment.

## 6. The Role of IL-9 in Combination Therapies including PD-1 Blockade

Several therapeutic approaches have shown a promising synergy with PD-1 blockade in preclinical studies and are being currently evaluated in clinical studies. Some of these therapies have been found to promote T_H_9 differentiation and IL-9 production. We speculate that IL-9 can contribute to the anticancer effects induced by combination therapies including PD-1 blockade, as discussed further below.

Combinations of PD-1 blockade with activation of co-stimulatory pathways in T cells represent a promising approach to induce an effective anti-tumor response. Co-stimulatory receptor glucocorticoid-induced TNFR-related protein (GITR) is widely expressed in cancer and its ligands induced anti-tumor responses in pre-clinical models. GITR agonists have been investigated in several clinical trials: monotherapy with GITR agonists exhibited limited efficacy, but combination therapies with PD-1 blockade showed encouraging results [62]. While stimulation with GITR ligands promoted expansion and effector functions of both CD4 and CD8 TILs [63], T_H_9 responses further contribute to their anti-tumor activity. Kim et al. showed that the anti-tumor effect of agonistic anti-GITR antibodies in several models of transplanted and autochthonous murine tumors was in large part mediated by IL-9 [64]. Treatment of tumor-bearing mice with an agonistic anti-GITR antibody induced T_H_9 cells and the efficacy of the treatment was remarkably reduced with the blockade of IL-9. Cytotoxic T lymphocyte (CTL) responses induced by the treatment in tumor-bearing mice were also markedly reduced in the absence of IL-9. These results indicated that the GITR agonist exerted its anti-tumor activity by promoting T_H_9 cell differentiation and following the enhancement of CTL responses. In addition to mouse models, the authors demonstrated that agonistic anti-human GITR antibody enhanced IL-9 expression in human T_H_9 cells. Because of the PD-1-driven regulation of T_H_9 cell function, combined GITR/anti-PD-1 immunotherapy may induce T_H_9 cell responses resulting in potent anti-tumor effects.

OX40 is another co-stimulatory pathway showing promising results in combination with PD-1 blockade. Like GITR, OX40 (other names include TNFRS4, CD134) is a member of the tumor necrosis factor receptor superfamily expressed on T cells in cancer. Activation of the OX40 receptor elicited potent anti-tumor responses in different models of solid tumors and monoclonal anti-OX40 antibody is now being assessed in early clinical trials [65]. Striking results were achieved in a mouse model of pancreatic ductal adenocarcinoma: while PD-1 blockade failed to induce durable anti-responses, combination anti-PD-1/OX40 therapy resulted in total tumor regression in treated mice [66]. Although more highly expressed in CD4 T cells, OX40 engagement promoted both CD4 and CD8 T cell responses in cancer patients [65]. In addition to promoting T cell activation, expansion, and memory cell formation, OX40 can shape the differentiation of CD4 T cells. OX40L-transgenic mice showed spontaneously induced T_H_9 cell responses associated with lung inflammation [67]. Consistently, OX40 signaling strongly promoted T_H_9 cell differentiation in vitro. This effect depended on non-canonical NF-κB signaling and the direct binding of p52 to the *Il9* promoter and did not depend on the transcription factors PU.1 and IRF4. In addition to promoting T_H_9 cells, OX40 signaling in CD4 T cells inhibited the differentiation of Foxp3^+^ Tregs and T_H_17 cells. These three CD4 T cell lineages rely on TGFβ signaling during their development; OX40 activation might thus favor the development of T_H_9 responses over T_H_17 and Treg responses in the presence of TGFβ. Depending on the cytokine environment, the combination of OX40 activation with PD-1 blockade may synergize in eliciting anti-tumor T_H_9 cell responses.

An additional approach designed to promote anti-PD-1-driven anti-tumor T cell responses lies in targeting “innate” immune pathways by activating pattern recognition receptors. Activation of several of these receptors in T cells enhances T_H_9 differentiation. cGAS-STING is a crucial pathway involved in anti-viral protection [68]. In addition to microbial DNA, the cGAS-STING pathway can be engaged by the host-derived DNA and drive spontaneous and therapy-induced immune responses in tumors [69,70]. The STING ligand 2′3′ cyclic GMP-AMP (cGAMP) synergized with PD-1/PD-L1 blockade in eliciting potent CTL responses and inhibiting tumor growth [71,72]. Based on preclinical data, several human STING agonists were developed and are currently being assessed as cancer therapeutics in clinical trials [73]. In addition to its well-acknowledged activity in inducing type I IFN production by myeloid cells, the STING pathway can modulate tumor immunity by regulating the differentiation of CD4 T cells. We demonstrated that intra-tumoral administration of the STING agonist cGAMP increased IL-9 and IFNγ expression in TILs [74]. Strikingly, the anti-tumor activity of cGAMP in mouse colon cancer and melanoma was impaired in the absence of CD4 T cell-derived IL-9 or IFNγ. Thus, T_H_9 together with T_H_1 cells represent important effector cell types mediating the anti-tumor activity of STING ligands. Further investigations are warranted to test whether these effector cytokines also control the efficacy of the combination of anti-PD-1 along with STING ligands.

Activation of Toll-like receptors (TLRs) promotes anti-tumor immunity and is used in cancer therapy: agonists for TLR2/4, TLR4, and TLR7 were approved by the FDA [75]. TLR2 ligands can also improve the outcomes of anti-PD-1 treatment. Activation of TLR2 enhanced anti-tumor CD8 T cell responses and boosted the efficacy of PD-1/PD-L1 blockade in murine melanoma [76]. While its role in DC activation has been acknowledged, TLR2 activation can deliver a direct co-stimulatory signal to T cells and furthermore shape the differentiation of CD4 T cells. TLR2 engagement increased IL-9 production in murine and human Th9 cells [77]. In non-polarizing conditions, co-stimulation of CD4 T cells through the TLR2 receptor increased IL-9 expression compared with classical CD28-driven co-stimulation without significantly affecting other lineage-specific CD4 T cell cytokines. TLR2 ligands may thus synergize with anti-PD-1 therapy by promoting T_H_9 cell responses in cancer.

## 7. Conclusions

Anti-PD-1 treatment has transformed cancer therapy by inducing unprecedented durable responses. However, low response rates warrant further research toward a more comprehensive understanding of the determinants of the response to this therapy. IL-9 has recently emerged as a cytokine associated with the response to the anti-PD-1 treatment. The studies in cancer patients who underwent nivolumab treatment and preclinical studies allow suggesting that PD-1 blockade induces IL-9 responses in tumors which in turn promote CD8 T cell functions (Figure 1). Different studies confirmed that the PD-1 axis can regulate IL-9 production in different IL-9-producing cells including T_H_9 cells and ILC2s. Further research is required to examine the contribution of different IL-9-producing immune cell types in the TME rescued by PD-1 blockade to the observed anti-tumor response.

IL-9 can synergize with PD-1 blockade in eliciting an anti-tumor immune response as demonstrated by recent findings [36]. Therefore, boosting IL-9 responses may give a therapeutic benefit and enhance response to PD-1 blockade. In addition to IL-9, promoting T_H_9 differentiation may be considered in combination with anti-PD-1 therapy. It would induce additional T_H_9-driven mechanisms such as enhancing CD8 T cell function in an IL-21-dependent manner and direct cytotoxicity against tumor cells. Several strategies are currently explored in clinical trials to overcome the resistance to anti-PD-1 treatment. Further investigations into the role of T_H_9 cells in the anti-PD-1-driven response may uncover novel therapeutic targets and biomarkers.

## Figures and Tables

**Table 1:** The role of IL-9 in human solid tumors.

Tumor Type (Number of Patients)	Location of IL-9 Expression	IL-9- Producing Cell Types	Correlation of IL-9 Expression with Survival	Effects of IL-9 on Tumor Cells and Immune Cells	Reference
NSCLC (32)	Malignant pleural effusion Blood	CD4	Negative correlation with OS	Promotes proliferation, survival, and migration of tumor cells (in vitro)	[17]
Metastatic melanoma (8)	TME Blood	CD4	Not investigated	Not investigated	[22]
Colitis- associated colorectal cancer (12)	TME	Immune cells, tumor cells	Not investigated	Promotes proliferation of tumor cells (in vitro)	[27]
Breast cancer (12)	TME Blood	CD4	Not investigated	Promotes cytotoxic capacity of CD8 T cells (ex vivo)	[21]
NSCLC (36)	TME	CD4	Positive correlation with RF survival	Not investigated	[25]
Cervical squamous cell carcinomas (28)	TME	CD4	Not investigated	Promotes MHC I expression in tumor cells, inhibits proliferation and survival of tumor cells (in vitro)	[28]
Endometrial carcinoma (143 + genomic data of 1274 patients)	TME	ILC2s, Vδ2 γδT cells, mast cells, macrophages, and T_H_9 cells	Positive correlation with OS	Correlates with higher tumor cell differentiation	[29]
Colorectal carcinoma (20)	TME Blood	CD4	Not investigated	Correlates with CD8 T-cell infiltration Correlates with high PD-1 expression	[26]
Muscle-invasive bladder cancer (259)	TME	Immune cells	Negative correlation with OS and RF survival	Correlates with high PD-1 expression and low granzyme and perforin expression in CD8 T cells	[30]
Renal clear cell carcinoma (66 + 537 patients from TCGA)	TME	Not investigated	Positive correlation with OS	Correlates with high tumor cell differentiation and lower (better) pathological score	[31]
Gastric cancer (453)	TME	Immune cells	Positive correlation with OS	Correlates with CD8 T-cell infiltration and expression of granzyme, perforin, and IFNγ by CD8 T cells Enhances tumor-killing capacity of CD8 T cells (ex vivo)	[32]
NSCLC (63)	TME Blood	CD4: T_H_9 and Treg, tumor cells	Not investigated	Correlates with low IFNγ and TNFα expression and high IL-21 expression	[33]

NSCLC—Non-Small Cell Lung Cancer, OS—Overall Survival, TME—Tumor Microenvironment, RF—Recurrence-Free, ILC2—innate lymphoid cells type 2, TCGA—The Cancer Genome Atlas.

**Table 2:** The role of IL-9 in the response to cancer immunotherapy.

Cancer Type (Number of Patients)	Location of IL-9 Expression	IL-9- Producing Cell Types	Correlation of IL-9 with Therapy Response	Effects of IL-9 on Anticancer Immunity	Reference
Metastatic melanoma (46)	TME Blood	CD4	Positive correlation with response to nivolumab	Co-localizes with CD8 T cells in tumors	[34]
Metastatic melanoma (76)	Blood	Not investigated (serum levels measured)	Positive correlation with response to TIL ACT	Not investigated	[35]
Muscle-invasive bladder cancer (259)	TME	Not investigated	Not investigated	Correlates with expansion and cytotoxic function of CD8 T cells in response to nivolumab	[30]
Gastric cancer (453)	TME	Immune cells	Not investigated	Synergizes with anti-PD-1 in promoting cytotoxic activity of CD8 T cells against tumor cells (ex vivo)	[32]

TIL—Tumor-Infiltrating Lymphocytes, ACT—Adoptive Cell Therapy.

## Data Availability

No new data were created or analyzed in this study. Data sharing is not applicable to this article.
